# Endometrial carcinoma in Kenya: clinical and biomarker profiles of 123 cases seen at two tertiary referral centers

**DOI:** 10.3389/fmed.2025.1607693

**Published:** 2025-09-10

**Authors:** Olivia Chesikaw, Allan Njau, Erick Chesori, Khadija Warfa, Anisa Mburu, Afrin Fatima Shaffi, Natalie Banet, Jonathan Wawire

**Affiliations:** ^1^Department of Anatomic Pathology, Aga Khan University, Nairobi, Kenya; ^2^Moi Teaching and Referral Hospital, Eldoret, Kenya; ^3^Aga Khan Hospital, Mombasa, Kenya; ^4^Cleveland Clinic, Cleveland, OH, United States

**Keywords:** endometrial carcinoma, hormone receptor biomarkers, Kenya, p53, mismatch repair proteins

## Abstract

**Introduction:**

Endometrial carcinoma (EC) is the second-most common gynecologic malignancy globally after cervical cancer, with varying incidence and outcomes across different populations. It comprises a heterogeneous group of tumors with distinct histopathologic and molecular characteristics. This study aims to provide a comprehensive clinicopathologic characterization of EC in the Kenyan population, focusing on histologic types and immunohistochemical biomarker expression.

**Methods:**

This retrospective study was conducted on archival tissue blocks from 123 patients diagnosed with EC at two major referral hospitals in Kenya. A tissue microarray block was constructed, and immunohistochemistry for 11 biomarkers, including ER, PR, p16, p53, NapsinA, ARID1A, PTEN, and Mismatch repair (MMR) proteins, was performed. Data analysis included descriptive statistics with Fisher’s exact test performed to compare proportions, while differences in means were compared using the Mann–Whitney U-test, with p < 0.05 considered significant.

**Results:**

The median age at diagnosis was 63 years, with the most common histologic type being endometrioid carcinoma (55.2%), followed by serous carcinoma (26%). Most low FIGO-stage patients had low-grade endometrioid histology. Obesity was the most frequently reported risk factor. Though hormone receptor biomarkers and p16 showed heterogeneous staining across all histologic types, ER and PR showed more frequent strong expression associated with endometrioid histology, while strong diffuse p16 staining was observed more frequently in serous carcinoma. MMR protein loss was predominantly observed in endometrioid carcinoma (30.0%), while all cases of serous carcinoma showed aberrant p53 expression. Abnormal p53 expression was also observed in 53.5% of all ECs, pointing to a higher percentage of patients with poor prognostic factors.

**Discussion:**

This study provides a comprehensive clinicopathologic and immunophenotypic profile of EC in a Kenyan population. The predominance of endometrioid carcinoma and the frequent loss of MMR proteins in this subgroup align with global trends. However, the high proportion of p53-abnormal tumors, particularly among serous carcinomas, suggests a significant presence of biologically aggressive disease. These findings highlight the utility of IHC as a practical and informative tool for subtype classification and risk stratification in resource-limited settings.

## Introduction

Endometrial carcinoma (EC) is the second most frequently diagnosed gynecologic malignancy, with rising incidence associated with increasing global rates of obesity (1). EC encompasses a heterogeneous group of tumors, with distinct histopathologic and molecular characteristics that influence prognosis and treatment strategies. The World Health Organization (WHO) classifies EC into seven histologic types: endometrioid carcinoma (EEC), serous carcinoma (SC), clear cell carcinoma (CCC), carcinosarcoma, mixed carcinoma, mesonephric carcinoma, and undifferentiated carcinoma (2). Among these, EEC is the most prevalent, particularly in high-income countries, accounting for approximately 75–90% of cases. Serous carcinoma, while less common, is noted for its aggressive clinical behavior and poorer prognosis (3).

Immunohistochemistry (IHC) plays a crucial role in the diagnostic workup of EC, aiding in the differentiation between histologic types and identification of molecular alterations that inform prognosis and guide treatment decisions. The molecular subtyping of EEC, which includes POLE-ultramutated, MMR-deficient, p53-mutant, and no specific molecular profile (NSMP), has provided significant insights into the pathogenesis and potential therapeutic targets for these tumors (4). Recent studies have also highlighted the importance of further characterizing EC with prognostic and potentially therapeutic biomarkers such as estrogen receptor (ER), progesterone receptor (PR), p53, and mismatch repair (MMR) proteins (5).

This study aims to provide a comprehensive clinicopathologic characterization of EC patients at two large referral hospitals in Kenya, with a focus on histologic types and the expression of key biomarkers by immunohistochemistry (IHC). The findings are expected to fill a significant gap in the existing literature, as there is limited data on the clinical and histologic profiles of EC from sub-Saharan Africa. The study will also evaluate the utility of IHC in the diagnostic and prognostic assessment of EC in this setting, where access to molecular testing is limited. By identifying the prevalence of specific histologic types and their associated immunohistochemical biomarker profiles, this research could inform local clinical practice and contribute to the development of tailored therapeutic strategies for patients with EC in Kenya.

## Materials and methods

### Study site

This was a retrospective analytical study conducted on archival tissue blocks (formalin fixed paraffin embedded, FFPE) of patients diagnosed and followed up at the Aga Khan University Hospital, Nairobi (AKUHN), a private tertiary teaching hospital in Kenya’s capital city of Nairobi, and Moi Teaching and Referral Hospital (MTRH), the largest public tertiary referral hospital located in Eldoret, serving majority rural populations of the Northern and Western Kenya. The study was conducted with approval from the Institutional Ethics Review Committee of the Aga Khan University, Moi University/MTRH, and the National Commission for Science, Technology and Innovation (NACOSTI). Construction of two tissue micro-array blocks was performed at a research core facility affiliated with the Dana-Farber/Harvard Cancer Center in Boston, United States, collaborating site, Massachusetts General Hospital, with approval for transfer of tissue blocks, granted by the Ministry of Health, Kenya. All samples were de-identified before transfer and analysis. There was no change of diagnosis after review, and neither the patients nor their physicians were contacted.

### Case selection

The samples were drawn from cases of EC diagnosed at the two sites between 2012 and 2022 from biopsy and resection specimens. A search and review of patient charts in the hospital health records was conducted. The biodata (age, sex) and clinical information (BMI, FIGO stage, treatment, and outcome, where available) were collected from the patient charts. Cases with clinical information and corresponding archived FFPE tissue blocks with adequate tissue for TMA construction were included in the study.

An initial 166 cases were identified, with 43 cases excluded due to lack of adequate tissue for further analysis, missing tissue blocks, or diagnosis other than EC on consensus review. A final patient population of 123 patients was analyzed.

A consensus review of hematoxylin and eosin (H&E) stained slides on all 123 cases by four pathologists (JW, AN, OC, EC, one of whom was a gynecologic pathologist (JW)) was then carried out to ascertain histopathologic findings (histologic type, histologic grade, myometrial invasion status, lymphovascular invasion, FIGO stage, where applicable). Histologic types were assigned after morphological assessment and review of immunohistochemical markers, where available. Confirmation of histologic type was completed on review of immunohistochemical markers performed on the tissue microarray described.

### Immunohistochemistry

A tissue microarray block was constructed by including two 1 mm cores from all eligible cases, with 25 controls at regular intervals. H&E and IHC for 11 biomarkers (ER, PR, P16, p53, MLH1, MSH2, MSH6, PMS2, ARID1A, PTEN, and Napsin A) were performed on 4 μm sections from the TMA block. Automated staining was performed using Ventana Benchmark Ultra HD (Roche) according to standard protocol. Immunohistochemistry was independently scored by three pathologists (JW, AN, OC) using the criteria shown in Table 1, with consensus scoring performed for discordant cases. A composite scoring system based on the Allred score (6), shown in Table 2, was utilized for scoring ER and PR, while a novel p16 composite score, shown in Table 3, was utilized for scoring p16.

**Table 1:** Summary of immunohistochemistry scoring.

Antibody	Clone	Vendor	IHC scoring
ER	SP1	Ventana	Allred Score (See Table 2)
PR	1E2	Ventana	Allred Score (See Table 2)
p16	E6H4	Ventana	p16 Composite Score (See Table 3)
p53	DO-7	Ventana	Null, Wild-type, Overexpressed (>75%)
PTEN	SP218	Ventana	Clonal loss, Complete loss, Retained
ARID1A	BAF250a	Sigma	Clonal loss, Complete loss, Retained
MLH1	M1	Ventana	Clonal loss, Complete loss, Retained
MSH2	G219-1129	Ventana	Partial loss, Complete loss, Retained
MSH6	SP93	Ventana	Partial loss, Complete loss, Retained
PMS2	A16-4	Ventana	Partial loss, Complete loss, Retained
Napsin A	MRQ-60	Ventana	Positive, Negative

**Table 2:** ER and PR composite (Allred) score.

Percentage of positive cells	Score
No staining	0
<1%	1
1–10%	2
10–33%	3
33–66%	4
>66%	5
Intensity of staining	Score
None	0
Weak	1
Moderate	2
Strong	3
Total Score	8

**Table 3:** p16 composite score.

Percentage of positive cells	Score
No staining	0
1–10%	1
11–20%	2
21–30%	3
31–40%	4
41–50%	5
51–60%	6
61–70%	7
71–80%	8
81–90%	9
91–100%	10
Pattern of expression	Score
No Staining	0
Focal	1
Diffuse	2
Intensity of staining	Score
No staining	0
Weak	1
Moderate	2
Strong	3
Total score	15

### Statistical analysis

Data was entered into a Microsoft Excel spreadsheet in a password-protected cloud storage, with analysis conducted on Stata version 16 (StataCorp LLC, College Station, TX). Descriptive statistics (proportion, mean, median) were used to present clinical and demographic parameters, frequencies of the various histologic types, and immunohistochemical profiles. Patients without recurrence were censored at the last date of being seen alive without recurrence. Fisher’s exact test was performed to compare proportions, while differences in means were compared using the Mann–Whitney U-test, with *p* < 0.05 considered significant.

**Figure 1:**
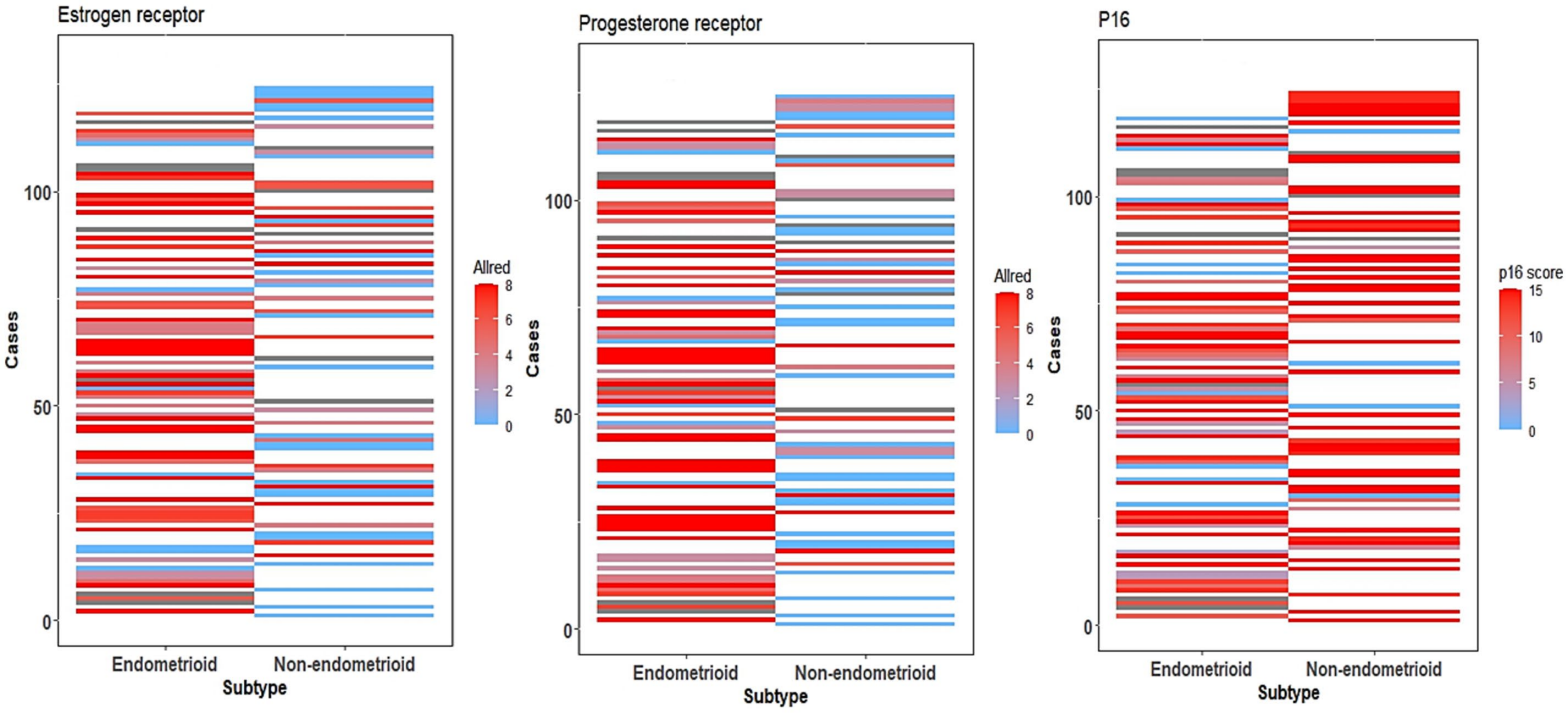
ER, PR, and p16 expression heatmaps. Heatmap showing ER, PR, and p16 expression of all cases using Allred (ER, PR) and p16 composites scores, comparing cases of endometrioid and non-endometrioid carcinoma.

To improve readability and avoid redundancy, we have summarized overlapping biomarker expression trends across histologic subtypes. Key comparative insights are retained and supplemented by visual data (Table 4; Figure 1) instead of detailed repetition across each biomarker section.

**Table 4:** A summary of clinical and immunohistochemical profiles of endometrial carcinoma.

		Low grade endometrioid (*n* = 50)	High grade endometrioid (*n* = 18)	Serous carcinoma (n = 32)	Carcinosarcoma (*n* = 15)	Others (clear cell, mixed, undifferentiated; *n* = 8)	Total (*n* = 123)
Median AGE		63 (*n* = 50)	65 (*n* = 18)	63.5 (*n* = 32)	65 (*n* = 15)	63 (*n* = 8)	**63 (*n* = 123)**
Median BMI		31.95 (*n* = 37)	28.0 (*n* = 12)	27.9 (*n* = 24)	28.0 (*n* = 10)	31.4 (*n* = 6)	**30.3 (*n* = 89)**
FIGO stage	Early stage FIGO stage I/II	33 (86.8%) (*n* = 38)	9 (64.3%) (*n* = 14)	12 (48.0%) (*n* = 25)	7 (70.0%) (*n* = 10)	3 (37.5%) (*n* = 8)	**64 (67.4%) (*n* = 95)**
	Late stage FIGO stage II/IV	5 (13.2%) (*n* = 38)	5 (35.7%) (*n* = 14)	13 (52.0%) (*n* = 25)	3 (30.0%) (*n* = 10)	5 (62.5%) (*n* = 8)	**31 (32.6%) (*n* = 95)**
ER	Positive	41 (89.1%) (*n* = 46)	13 (86.7%) (*n* = 15)	17 (62.9%) (*n* = 27)	6 (40.0%) (*n* = 15)	2 (25.0%) (*n* = 8)	**79 (*n* = 111)**
	Average composite score	5.8	5.0	3.9	2.0	1.1	**-**
PR	Positive	41 (91.1%) (*n* = 45)	13 (86.7%) (*n* = 15)	14 (51.9%) (*n* = 27)	5 (33.3%) (*n* = 15)	2 (25.0%) (*n* = 8)	**75 (*n* = 110)**
	Average composite score	6.1	4.9	2.7	1.6	1.4	**-**
P16 average composite score (out of 15)		9.2 (*n* = 46)	11.2 (*n* = 15)	13.1 (*n* = 29)	12.9 (*n* = 15)	11.0	**(*n* = 105)**
P53 mutated pattern		9 (20.9%) (*n* = 43)	9 (64.3%) (*n* = 14)	28 (100%) (*n* = 28)	10 (71.4%) (*n* = 14)	4 (50.0%) (*n* = 8)	**60 (56.1%) (*n* = 107)**
Loss of any MMR protein expression		13 (28.9%) (*n* = 45)	5 (33.3%) (*n* = 15)	0 (0%) (*n* = 29)	1(<1%) (*n* = 14)	1 (12.5%) (*n* = 8)	**20 (18.0%) (*n* = 111)**
PTEN loss		26 (61.9%) (*n* = 42)	10 (71.4%) (*n* = 14)	11 (37.9%) (*n* = 29)	8 (57.1%) (*n* = 14)	5 (62.5%) (*n* = 8)	**60 (56.1%) (*n* = 107)**
ARID1A loss		11 (23.9%) (*n* = 46)	3 (20.0%) (*n* = 15)	3 (11.1%) (*n* = 27)	2 (13.3%) (*n* = 15)	0 (0%) (*n* = 8)	**19 (17.1%) (*n* = 111)**

Values in bold indicate number of cases.

## Results

### Patient characteristics

The study had a population of 123 patients, 92 from AKUHN and 31 from MTRH. The patients were aged between 34 and 90 years, with a median age of 63 years. The study reviewed sections from archived FFPE blocks obtained from 95 hysterectomy samples and 55 biopsy samples. A total of 57 patients had both biopsy and hysterectomy samples available for review.

Data on BMI were available for 89 patients, with a median BMI of 30.3 Kg/m^2^. The highest median BMI was observed in cases of low-grade EEC (31.95 Kg/m^2^) and the lowest in serous carcinoma (27.9 Kg/m^2^). There were no significant differences between the endometrioid and non-endometrioid histologic types (*p* = 0.23).

Pathological (FIGO) staging information was available for 95 of the 123 patients. Early-stage (FIGO I or II) prevalence was highest in low-grade EEC (86.8%), with serous carcinoma having the highest late-stage (FIGO stage III or IV) prevalence (52.0%). In total, 64 patients (67.4%) were in the early stage, of whom 42 (65.6%) showed endometrioid histology. Significantly, of the 31 patients with a high stage at diagnosis, 21(49%) showed non-endometrioid histology (*p* = 0.004).

### Histologic types and immunohistochemical profiles

Six histologic subtypes of EC were identified. Sixty-eight patients (55.2%) showed endometrioid histology, of which 50 cases were low-grade (FIGO grade 1 and 2) and 18 were high-grade (FIGO grade 3). Fifty-five patients (44.8%) showed non-endometrioid histology with frequencies distributed as follows: serous, 32; carcinosarcoma,15; clear cell,5; mixed carcinoma, 2; and dedifferentiated carcinoma, 1. The clinical, demographic, and immunohistochemical profiles of these cases are summarized in Table 4.

ER expression was evaluated in 111 cases, using a composite scoring system based on the Allred score, with expression patterns summarized in Figure 1. The highest expression was seen in low-grade EEC (89.1%) with an average composite score of 5.8, followed by high-grade EEC (86.9%). Notably, 63.5% of serous carcinomas showed ER expression with an average composite score of 3.9 and had no significant difference when compared to high-grade EEC (*p* = 0.15798).

PR expression, as with ER, was evaluated in 110 cases, with expression patterns summarized in Figure 1. The highest rate and composite scores were seen in low-grade EEC (91.1%, 6.1), followed by high-grade EEC (86.7%, 4.9). However, unlike ER, PR expression was more likely in high-grade EEC when compared to serous carcinoma (*p* = 0.04229).

**Figure 2:**
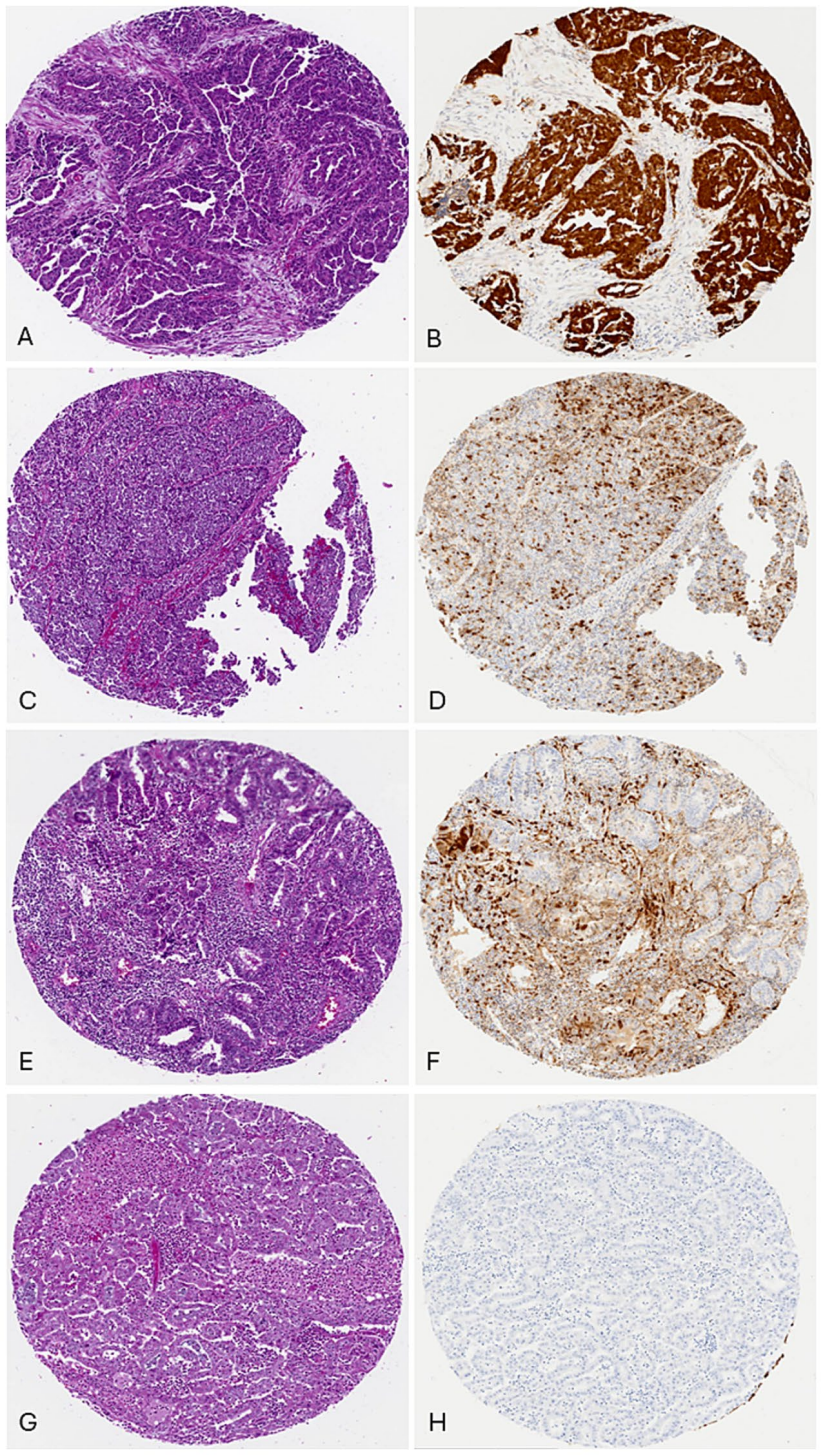
p16 Composite scoring: **(A)** case 1, uterine serous carcinoma, H/E. **(B)** p16 scores: percentage: 91–100% (10), intensity: strong (3), pattern: diffuse (2), total score: 15/15. **(C)** case 69, Endometrial endometrioid carcinoma, FIGO grade 3, H/E. **(D)** p16 scores: percentage: 31–40% (4), intensity: strong (3), pattern: focal (1), total score: 7/15. **(E)** case 45, endometrial endometrioid carcinoma, FIGO grade 1, H/E. **(F)** p16 scores: percentage: 11–20% (2), intensity: moderate (2), pattern: focal (1), total score: 5/15. **(G)** case 37, endometrial endometrioid carcinoma, FIGO grade 1, H/E. **(H)** p16 scores: Percentage: 0% (0), intensity: not applicable (0), pattern: not applicable (0), total score: 0/15.

Of the 105 cases evaluated for p16 expression using a composite scoring system summarized in Table 3, with results exemplified by photomicrographs in Figure 2. The highest average composite scores were seen in serous carcinoma (13.1) and the lowest in low-grade EEC (9.2). The diffuse, strong p16 expression pattern was also noted in high-grade EEC, which had an average composite score of 11.2, comparable to that seen in serous carcinoma (*p* = 0.25848). Overall, however, strong diffuse p16 staining was associated with non-endometrioid histologic types as depicted in the expression patterns in Figure 1.

A total of 111 cases were evaluated for expression of 4 MMR proteins (MLH1, PMS2, MSH2, MSH6). Twenty-two cases showed clonal or complete loss of at least one protein. The highest prevalence of loss of any protein was observed in EEC, with 18 of 60 cases (30.0%), 12(66.7%) of which showed concurrent loss of MLH1 and PMS2, the most prevalent combination lost. Overall, MMR loss was more commonly observed in endometrioid histology compared to serous carcinoma (*p* = 0.0013).

One hundred and seven cases were evaluated for expression of p53, of which a total of 60 (56.1%) cases showed a mutated pattern (54 overexpressed, 6 null). All 28 cases of serous carcinoma for which p53 was interpreted showed a mutated p53 pattern (24 over-expressed, 4 null), while a total of 9 cases (20.9%) of low-grade EEC also showed mutated patterns (8 overexpressed, 1 null). Two of these cases showed loss of at least one MMR protein. All 9 other cases of high-grade EEC with p53 mutated pattern were MMR proficient (8 overexpressed, 1 null). Cytoplasmic staining for p53 was not observed in any of the cases. Overall, when compared to EEC, p53 mutated patterns were more likely observed in serous carcinoma (*p* = 0.0002), even when serous carcinoma is compared only to high-grade EEC (*p* = 0.0008).

Of the 107 cases evaluated for PTEN expression, the highest prevalence of complete or partial PTEN loss was seen in EEC (36 cases, 64.3%), with 71.4% (10 cases) of high-grade EECs showing partial or complete loss of the protein compared to serous carcinoma that showed loss in 11 cases (37.9%; *p* = 0.05461). PTEN was lost in 12 of 17 cases (70.6%) of EEC with MMR loss.

ARIDA1 expression was evaluated in 111 cases, with the highest prevalence of complete or partial loss seen in low-grade EEC (11 cases, 23.9%). A total of 14 cases of EEC (23.0% of cases) showed loss of ARIDA1, 5 of which showed concurrent loss of at least one MMR protein, while 3 demonstrated abnormal p53 expression. There was no significant difference between serous carcinoma with a loss rate of 11.1% and high-grade EEC at 20.0% (*p* = 0.64871).

A total of 112 cases were evaluated for the expression of Napsin A, of which expression was observed in 12 cases, including 3 cases of clear cell carcinoma. Two cases of high-grade EEC showed Napsin A expression (one case was positive for ER and PR, while the second, though negative for ER and PR, showed p53 overexpression and loss of MLH1, PMS2, and PTEN). Of the 5 cases of serous carcinoma that showed Napsin A expression, 2 were positive for ER and PR, while the other 3 showed diffuse, strong p16 expression, like the 1 case of carcinosarcoma.

### Outcome and survival

Lymphovascular space invasion (LVSI) status was assessed during histopathologic review; however, no statistically significant association was observed between LVSI and biomarker expression (including p53 mutation pattern or MMR protein loss). This may be due to limited sample size and missing LVSI data in some cases. Nevertheless, given LVSI’s known prognostic impact, especially in high-grade tumors, its correlation with molecular subtypes remains an important area for future investigation in larger cohorts.

Losses to follow-up severely limited our follow-up data. Overall, this data was available for only 26 patients with a median follow-up period of 29 months (range 1–102 months). Nineteen of these 26 patients were still alive at the end of the study period, with two reported recurrences at 18 and 20 months. Notably, of the 7 patients who had died, 6 were of non-endometroid histology (carcinosarcoma 3, serous 2, mixed carcinosarcoma 1).

## Discussion

In this study, morphologic assessment aided by immunohistochemistry was used on biopsy or hysterectomy samples from 123 patients seen at two major tertiary referral centers in Kenya to classify EC in the Kenyan population. The findings will inform local practice and research in screening and diagnosis, including the diagnostic, therapeutic, and prognostic value of immunohistochemistry.

The median age at diagnosis for our study population was 63 years, with over 95% of the patients above the age of 50. There were no significant differences in age across the various histologic types. Interestingly, this finding is similar to reports from studies conducted in higher-income countries (1, 3), despite contrasting demographic profiles and risk factors between the populations represented. Data on parity, age at menarche, contraceptive use, and family history of malignancy were largely missing from the records, preventing a comprehensive evaluation of other risk factors.

Overall, six histologic types were identified in our study population, with EEC being the most frequent (68/123 cases, 65.6%), followed by serous carcinoma (32 cases, 26.0%). Serous carcinoma accounts for a much larger proportion of patients in our study compared to findings reported from populations in higher and middle-income countries, where endometroid type accounts for up to 90% of cases (1, 3). It has, however, been suggested, by studies in the United States, that serous carcinoma is seen more frequently in Black women than their white counterparts (7, 8), raising the question as to whether this could play a role in the distributions noted in the current study.

Obesity is not only a strong modifiable risk factor for EC but is also associated with increased cancer recurrence (9). In our study, in the 87 patients for which BMI data were available, the median BMI at diagnosis was 30.3 Kg/m^2^, without a significant difference between endometrioid and non-endometrioid histological types. Though the highest median BMI was seen in patients with low-grade EEC (32 Kg/m^2^) and the lowest in those with serous carcinoma (27.9 Kg/m^2^), this difference was not significant. Furthermore, only 42.6% of patients with EEC were obese compared to 34% of patients with serous carcinoma. These findings highlight the need to better understand the risk factors driving the pathogenesis of EC in our population.

It is generally reported that at least half of patients with serous carcinoma have extrauterine spread at the time of diagnosis compared to patients with endometrioid histology (10). Our findings were consistent with these reports. FIGO staging information was available for 25 of 32 patients with serous carcinoma. Thirteen patients were diagnosed at an advanced stage (III or IV), reflecting the aggressive nature of this disease and probable delays in diagnosis. Meanwhile, 42 of the 64 patients (65.6%) with low-stage disease showed endometrioid histology, with 33 (78.6%) of these patients being low-grade EEC.

Immunohistochemistry is a necessary adjunct to diagnosis, particularly in the distinction between tumors with high-grade histology, where the differentials may include high-grade EEC, serous carcinoma, carcinosarcoma, and clear cell carcinoma. Morphologic diagnosis is especially prone to suboptimal interobserver variability (11). This limitation has hampered attempts to answer the question of whether grade 3 EEC is as aggressive as SC or CCC. To aid in this distinction, a panel of antibodies, including p53, p16, DNA mismatch repair proteins, PTEN, and ARID1A, has been suggested (12). Additionally, Napsin A may be used if CCC is in the differential diagnosis (12). In this study, this panel, in addition to hormone receptors ER and PR, was utilized to augment the morphologic diagnosis while assessing their patterns of expression across the various histologic types.

We found some overlap in the expression of these markers across the various histologic types. Hormone receptors, for instance, were expressed in more than 80% of EECs and 63% of serous carcinomas. These rates are like those reported in previous studies, where ER and PR were expressed in 80% of endometrioid tumors, up to 54% of serous carcinomas (13). A composite score, combining the percentage of positive cells and the strength of expression (strong, moderate, or weak) with a maximum score of 8, was employed to further define the pattern of expression. The highest average scores for both ER and PR were seen in low-grade endometrioid carcinoma (5.8 and 6.1, respectively), with the lowest scores seen in carcinosarcoma (2.7 and 1.7, respectively). When compared to serous carcinoma, however, high-grade EEC showed an ER composite score of 4.5 compared to 5.0 seen in serous. PR, however, was significantly more strongly and diffusely expressed in high-grade EEC (composite score of 4.8) than in serous carcinoma (3.0). These findings suggest value in considering the pattern of expression in the assessment of ER and PR in distinguishing between the various high-grade histologic types.

P16 is another important biomarker in the diagnosis of high-grade EC (12, 13). The extent of p16 expression should be considered in its analysis to aid the distinction between grade 3 EEC and serous carcinoma. This has been demonstrated by a study in which serous carcinomas showed moderate to strong diffuse p16 expression in 91–100% of cells compared to less intense and less diffuse staining in 11–90% of cells in grade 3 endometroid carcinoma (14). Once more, utilizing the composite score, we considered the percentage of cells positive, intensity of staining, and pattern of staining for a total score out of 15. Cases of serous carcinoma had the highest average composite score of 13.1, followed by carcinosarcoma with 12.9. Grade 3 EEC had an average composite score of 11.1, while the low-grade EEC had the lowest composite score of 10.0. P16 shows some degree of expression in all histologic types of EC. Its powers of distinction, however, lie in considering its pattern, extent, intensity, and proportion of cells.

MMR protein immunohistochemistry is important in identifying patients with microsatellite instability. MMR expression is used in the molecular classification of EEC, as loss of any protein is associated with improved overall and progression-free survival (15). This group of patients may also benefit from the addition of immunotherapy to their treatment. MMR proteins are also an essential diagnostic aid in distinguishing serous carcinoma from EEC, with the latter more likely to demonstrate loss of MMR proteins. Previous studies have demonstrated aberrant MMR expression in 57% of endometrioid carcinomas. MLH1 and PMS2 are reportedly the most affected proteins (involving 59% of cases) (16). Complete or partial loss of at least one MMR protein was reported in a lower proportion of cases in our study (30.0% of cases of EEC). Despite a lower proportion of MMR protein loss among cases of EEC, there remains utility in the distinction from serous carcinoma.

Abnormal p53 expression is often associated with serous carcinoma, as confirmed by our All 28 cases of serous carcinoma for which p53 was interpreted showed a mutated p53 pattern. It also carries significant prognostic weight in EEC, with the current molecular classification identifying p53-abnormal EC as the subgroup with the poorest prognosis and most likely to benefit from adjuvant therapy. Tumors with p53 mutations are almost always *POLEwt* with proficient MMR expression (17). Given the high sensitivity and specificity of p53 immunohistochemistry as a surrogate for next-generation sequencing, it is of great utility in settings like the study sites, where molecular testing is currently unobtainable (18). In this study, 18 cases (31.6%) of EEC showed a p53 mutated pattern (16 overexpressed, 2 null). Of these 18 cases,16 showed p53 overexpression without any loss of MMR expression, a high proportion of EEC with likely a poor prognostic factor, even in the absence of molecular testing for *POLE* mutations. Unlike other studies, we did not report a clonal loss pattern, as this was performed on a microarray.

ARID1A is a tumor suppressor gene whose mutations, characterized by loss of staining in tumor cells, are implicated in the pathogenesis of EEC. Loss of expression has been reported in 30–50% of cases of low-grade EEC and 40–60% of high-grade EECs (19). Cases of EEC with loss of ARID1A are also enriched for MMR deficiency and show a favorable prognosis (20, 21). In our study, 23% of EECs showed loss of ARID1A, 5 of which showed concurrent MMR deficiency. However, given the lower prevalence of ARID1A and MMR loss than reported elsewhere, this association was not statistically significant (*p* = 0.1096). In serous carcinoma, ARID1A loss has been reported in 9–14% of cases and has been suggested as a useful marker in the distinction between high-grade EEC and serous carcinoma (22, 23). Our study reports a relatively low frequency of ARID1A in high-grade EEC (20%) compared to serous carcinoma (11.1%), hence limiting its use in making this distinction (*p* = 0.64871).

PTEN is a critical tumor suppressor whose loss of function is frequently implicated in the pathogenesis of EEC and is characterized by loss of nuclear and or cytoplasmic expression by immunohistochemistry (24). PTEN loss has been demonstrated in 55–80% of EECs (25) and is frequently lost in MMR-deficient tumors, hence associated with a favorable prognosis (26). In our study, PTEN was lost in 64.3% of EECs and 70.6% of EECs with MMR loss, in comparison to SC, which showed PTEN loss in 11 cases (37.9%; *p* = 0.0237). This difference justifies the inclusion of PTEN in the immunohistochemical panel for the distinction between SC and high-grade EEC.

Previous studies have demonstrated Napsin A expression in 60–90% of CCC (27), a finding that is particularly useful in the distinction of CCC from SC or EEC with cytoplasmic clearing. In our study, out of 5 cases of CCC, 3 were positive for Napsin A, while 9 other positive cases were spread across the other histologic types. From a diagnostic standpoint, Napsin A may be of limited value if not included in a larger panel of antibodies for making the distinction between CCC and its morphologic mimics.

Numerous studies show significant prognostic differences between serous carcinoma and endometrioid carcinoma. This study was severely hampered by the paucity of follow-up data, limiting exhaustive analysis of clinical outcomes for the various histologic types. Given their relative proportions, however, it seems likely that since our study population had a higher proportion of non-endometrioid histologic types compared to populations in higher-income countries, it may translate to higher mortality for EC overall in this population despite a lower incidence rate. Additionally, a smaller proportion of our study cohort had aberrant MMR expression, which is often associated with better outcomes.

One of the limitations of this study is the relatively small sample size and lack of follow-up data, being a retrospective study, which limits the generalizability of the findings to the broader Kenyan population or other sub-Saharan African populations. Additionally, the study relied on immunohistochemistry (IHC) rather than molecular subtyping, which, while useful, may not capture the full spectrum of genetic alterations (such as POLE mutations) present in EC, particularly in resource-limited settings where advanced molecular testing is not feasible. The lack of long-term follow-up data also limits the ability to assess the prognostic impact of the histologic types identified. Future research should aim to include larger, multicenter cohorts to enhance the representativeness of the findings, incorporate advanced genomic techniques to provide a more comprehensive molecular characterization, and establish long-term follow-up protocols to better understand the clinical outcomes associated with different histologic and molecular subtypes of EC in this population.

In conclusion, this study demonstrates that endometrioid endometrial carcinoma (EEC) is the predominant histologic subtype in the Kenyan population, comprising 55.2% of cases, with low-grade EEC being the most frequent. High-grade subtypes, including serous carcinoma and carcinosarcoma, accounted for a significant proportion (44.8%) and were more frequently associated with advanced-stage disease. Low-grade EECs were typically diagnosed at early FIGO stages and were more likely to exhibit favorable biomarker profiles, including strong ER and PR expression, PTEN retention, and MMR deficiency. In contrast, high-grade tumors, particularly serous carcinoma, exhibited more aggressive features, including p53 mutations and strong diffuse p16 expression, with a lower prevalence of hormone receptor positivity. These distinctions underline important clinicopathologic differences between low-grade and high-grade EC and emphasize the critical role of immunohistochemistry in subtyping EC, especially in settings with limited access to molecular diagnostics.

## Data Availability

The original contributions presented in the study are included in the article/supplementary material, further inquiries can be directed to the corresponding author.
